# Editorial: EMG Signal Acquisition, Processing, and Analysis—Bridging the Gap Between Research and Daily Practice

**DOI:** 10.3390/s26082344

**Published:** 2026-04-10

**Authors:** Luca Miceli

**Affiliations:** Pain Therapy Department, Centro di Riferimento Oncologico, 33081 Aviano, Italy; luca.miceli@cro.it

## 1. Introduction

The field of surface electromyography (sEMG) has undergone a profound transformation over recent decades, evolving from a specialized laboratory tool to a versatile instrument widely utilized in rehabilitation, sports science, and occupational health. Initially confined to academic research for analyzing superficial muscle electrical activity, sEMG has benefited from technological advancements that extend its clinical applications, enabling non-invasive assessments of voluntary muscle contractions in dynamic settings. These innovations include high-density wearable sensors, improved signal processing algorithms to handle inter-subject variability, and integration with real-time biofeedback systems, making it accessible beyond research labs [1,2,3,4].

Recent studies have highlighted sEMG’s critical role in continuous monitoring for rehabilitation, such as post-surgical recovery and neurodegenerative disease management, where it enhances diagnostic precision and personalized therapy. In sports, sEMG supports biomechanical analysis and injury prevention by evaluating muscle activation patterns during complex movements like running or skating. Similarly, in occupational health, it detects ergonomic risks from load handling, identifying muscle fatigue and abnormal co-activations to prevent musculoskeletal disorders [5,6,7,8].

This transformation addresses longstanding challenges, including signal artifacts from motion and the need for robust normalization methods like MVC or submaximal contractions, ensuring reliability in daily practice. Wearable sEMG systems now facilitate ecological validity, bridging the gap between controlled experiments and real-world applications in diverse fields [9,10].

This Special Issue, entitled “EMG Signal Acquisition, Processing and Analysis: From Research to Daily Practice in the Rehabilitation, Sports, and Occupational Fields,” was conceived to explore the delicate balance between high-quality data acquisition and practical usability for non-researcher professionals.

## 2. Overview of the Published Papers

The 14 articles included in this collection highlight a significant shift toward more responsive, scalable, and intelligent systems that translate complex physiological signals into actionable insights for patients, athletes, and workers. Emerging Trends in AI and Deep Learning: A primary trend identified across the contributions is the integration of Advanced Machine Learning and Deep Learning (DL) architectures for motion and force estimation. Researchers are moving beyond traditional linear models to capture the complex, non-linear nature of sEMG signals. For instance, the combination of Temporal Convolutional Networks (TCN) with Convolutional Block Attention Modules (CBAM) has proven highly effective for multi-joint lower-limb angle estimation, enhancing feature extraction across different movement patterns like walking and squatting. The application of Transfer Learning (TL) has emerged as a crucial solution to the problem of limited dataset diversity. By leveraging models pre-trained on different muscle joints (e.g., from elbow to hand-wrist), it is now possible to achieve high accuracy in force estimation even with significantly reduced calibration data. Furthermore, innovative architectures like the Kolmogorov–Arnold Network (KAN) are pushing the boundaries of performance prediction, autonomously discovering latent electrophysiological patterns to forecast explosive actions, such as punching force in Wushu Sanda. Enhancing Real-Time Responsiveness and Fluency: Improving system responsiveness remains a cornerstone of modern human–machine interfaces. Several studies in this issue explore the utilization of Electromechanical Delay (EMD)—the physiological lag between electrical activation and physical motion—to create anticipatory control systems. By predicting intended movements 50 to 200 ms in advance, robotic agents and myoelectric prostheses can achieve higher “fluency,” reducing lag and making interactions feel more natural for the user. This is particularly vital in myoelectric prosthetics, where minimizing delays below 300 ms is essential for preventing user rejection. Methodological Rigor and Signal Integrity: As EMG analysis moves into daily practice, the issue of signal integrity and normalization remains paramount. This collection includes critical evaluations of how different procedures—such as Maximum Voluntary Contraction (MVC) versus remote voluntary contractions (RVC)—impact data reliability. Findings suggest that while MVC is the gold standard, RVC often provides a more stable reference for dynamic tasks, especially in clinical populations where maximal effort is difficult to achieve. Furthermore, the challenge of non-stationarity in EMG signals during dynamic movements has been addressed through advanced time–frequency (TF) analysis. The comparative evaluation of methods like Short-Time Fourier-Transform (STFT), Continuous Wavelet Transform (CWT), and Noise-Assisted Multivariate Empirical Mode Decomposition (NA-MEMD) provides a methodological roadmap for characterizing rapid spectral transitions. Such precision is essential for diagnosing neurodegenerative conditions, as demonstrated in animal models of Parkinson’s disease. Applications in Clinical Rehabilitation and High-Performance Sports: The practical applications showcased here span a wide array of activities:

- Neuromuscular Adaptations: Investigations into muscle synergy patterns (extracted via NMF) reveal how combined interventions, such as NMES and water-based resistance training, reorganize coordination for more efficient freestyle kicking in elite swimmers.
- Clinical Retraining: Research on post-stroke adults highlights the compensatory roles of intrinsic and functional stiffness, emphasizing the need for multidimensional evaluations of postural tone.
- Sport-Specific Biomechanics: Studies analyzed muscle recruitment in diverse contexts, from the impact of footwear on core and pelvic floor activation in female runners to asymmetries in flamenco footwork and training-specific patterns in roller speed skating.
- Explosive Performance: The synergistic effects of Neuromuscular Electrical Stimulation (NMES) were validated as an effective method for enhancing explosiveness in badminton jump smashes.
- Field Technology: Comparative studies between GPS-derived data and EMG in soccer players warn that established markers may underestimate the actual neuromuscular demands during resisted sprints.

## 3. Conclusions

Recent developments in sEMG have seen a pivotal shift toward integrating high-density wearable sensors with advanced Deep Learning architectures. However, a persistent gap remains, namely the difficulty in translating laboratory precision into generalizable, real-world tools, largely due to high inter-subject variability and the computational cost of processing non-linear dynamics. This Special Issue has addressed these gaps by validating Transfer Learning to mitigate data scarcity, employing Kolmogorov–Arnold Networks to capture complex electrophysiological patterns, and utilizing Electromechanical Delay to eliminate responsiveness lags in human–machine interfaces. Looking ahead, future research must move beyond discrete state classification toward high-resolution regression models for continuous multi-joint trajectory prediction. A primary frontier lies in developing hybrid frameworks that combine functional anatomy principles with transformer-based architectures to improve the fluency of myoelectric prosthetics and exoskeletons. Furthermore, longitudinal studies are essential to track the evolution of neuromuscular adaptations over time, particularly in neurodegenerative populations. Finally, researchers should prioritize optimizing these complex algorithms for edge computing on mobile hardware, ensuring that advanced sEMG analysis becomes ecologically viable and accessible for daily clinical and sports practice.

## References

[1] Farina D., Enoka R.M. (2023). Evolution of surface electromyography: From muscle electrophysiology towards neural recording and interfacing. J. Electromyogr. Kinesiol..

[2] Campanini I., Disselhorst-Klug C., Rymer W.Z., Merletti R. (2020). Surface EMG in Clinical Assessment and Neurorehabilitation: Barriers Limiting Its Use. Front. Neurol..

[3] Ranavolo A., Serrao M., Draicchio F. (2020). Critical Issues and Imminent Challenges in the Use of sEMG in Return-To-Work Rehabilitation of Patients Affected by Neurological Disorders in the Epoch of Human–Robot Collaborative Technologies. Front. Neurol..

[4] Etana B.B., Malengier B., Krishnamoorthy J., Van Langenhove L. (2024). Integrating Wearable Textiles Sensors and IoT for Continuous sEMG Monitoring. Sensors.

[5] Haab T., Leinen P., Burkey P. (2024). Role and effectiveness of surface EMG feedback in sports and orthopedic rehabilitation: A systematic review. Explor. Musculoskelet. Dis..

[6] Agostini V., Ghislieri M., Rosati S., Balestra G., Knaflitz M. (2020). Surface Electromyography Applied to Gait Analysis: How to Improve Its Impact in Clinics?. Front Neurol..

[7] Donisi L., Jacob D., Guerrini L., Prisco G., Esposito F., Cesarelli M., Amato F., Gargiulo P. (2023). sEMG Spectral Analysis and Machine Learning Algorithms Are Able to Discriminate Biomechanical Risk Classes Associated with Manual Material Liftings. Bioengineering.

[8] Gül S., Büyüklüoğlu G., Kaya İ., Özer O., Özgür E.M., Ülkar B.Ü. (2023). Surface electromyography as a follow-up instrument in anterior cruciate ligament reconstruction rehabilitation. Medicina Dello Sport.

[9] Ait Yous M., Agounad S., Elbaz S. (2025). Detection, identification and removing of artifacts from sEMG signals: Current studies and future challenges. Comput. Biol. Med..

[10] Gomez-Correa M., Cruz-Ortiz D. (2022). Low-Cost Wearable Band Sensors of Surface Electromyography for Detecting Hand Movements. Sensors.

